# Quantification of re-absorption and re-emission processes to determine photon recycling efficiency in perovskite single crystals

**DOI:** 10.1038/ncomms14417

**Published:** 2017-02-21

**Authors:** Yanjun Fang, Haotong Wei, Qingfeng Dong, Jinsong Huang

**Affiliations:** 1 Department of Mechanical and Materials Engineering, University of Nebraska-Lincoln, Lincoln, Nebraska 68588, USA

## Abstract

Photon recycling, that is, iterative self-absorption and re-emission by the photoactive layer itself, has been speculated to contribute to the high open-circuit voltage in several types of high efficiency solar cells. For organic–inorganic halide perovskites that have yielded highly efficient photovoltaic devices, however, it remains unclear whether the photon recycling effect is significant enough to improve solar cell efficiency. Here we quantitatively evaluate the re-absorption and re-emission processes to determine photon recycling efficiency in hybrid perovskite with its single crystals by measuring the ratio of the re-emitted photons to the initially excited photons, which is realized by modulating their polarization to differentiate them. The photon recycling efficiencies are revealed to be less than 0.5% in CH_3_NH_3_PbI_3_ and CH_3_NH_3_PbBr_3_ single crystals under excitation intensity close to one sun, highlighting the intrinsically long carrier recombination lifetime instead of the photon-recycling-induced photon propagation as the origin of their long carrier diffusion length.

## Abstract (web summary)

Fang *et al*. develop a method to determine the photon recycling efficiency for organic-inorganic hybrid single crystal perovskites by differentiating between emitted and re-absorbed photons based on their polarization difference. For these systems efficiencies of less than 0.5% are reported.

The power conversion efficiency (PCE) of organic–inorganic halide perovskite (OIHP)-based solar cells has skyrocketed from 3.8% to a certified 22.1% in less than six years^1^,^2^,^3^,^4^,^5^,^6^,^7^,^8^,^9^,^10^,^11^,^12^,^13^,^14^,^15^,^16^,^17^,^18^. This is already higher than that of the commercial multicrystalline Si solar cells and is approaching that of single crystal ones. One contribution of the high PCE of OIHP solar cells is from its small open-circuit voltage (*V*_OC_) deficiency, that is, (*E*_g_–*eV*_OC_)/*e*, where *E*_g_ is the optical band gap, and *e* is the elementary charge. We have demonstrated that by mitigating the energy disorder in the organic electron transport layer via solvent annealing, the *V*_OC_ deficiency of planar-structured CH_3_NH_3_PbI_3_ (MAPbI_3_) perovskite solar cells can be reduced to as low as 0.42 V. *Bi et al*.^19^ also reported that by optimizing the composition of the perovskite layer to enhance the electroluminescence yield of the solar cells, the *V*_OC_ of the best device with a PCE of 20.8% can reach 1.15 V. This corresponds to a *V*_OC_ deficiency of 0.40 V, which is much lower than that of organic solar cells and is comparable to those of copper indium gallium selenide (CIGS) and single crystal Si solar cells^20^. However, the present *V*_OC_ deficiency in OIHP solar cells is still larger than that of the best GaAs solar cells, which is around 0.30 V, while in the latter case it was proposed that the photon recycling effect contributes to the high *V*_OC_. So a hypothesis that naturally arises is that the photon recycling effect may also exist in OIHP materials giving rise to high *V*_OC_ in perovskite solar cells. There are two kinds of definitions of photon recycling in literature^21^,^22^. One only considers one-cycle of photon re-absorption, which refers to the radiatively emitted photons after external light excitation can be absorbed again by the photoactive layer itself. In this case, the photon recycling efficiency is the ratio of re-absorbed photons versus the total emitted photons. As shown in Fig. 1(a), for a 1 mm thick CH_3_NH_3_PbBr_3_ (MAPbBr_3_) single crystal, most of the photoluminescence (PL) spectrum falls in its own absorption spectrum, leading to a re-absorption ratio of 94.2% calculated by dividing the integrated intensity of the self-filtered PL by that of the original PL. The other definition considers the multiple cycles of photon re-emission and re-absorption processes, and thus the way to characterize the photon-recycling efficiency is to measure the final emission intensity after multiple cycles. As a result of the repeated re-absorption and re-emission processes, the photon recycling effect allows the building up of charge carriers in the active layer to increase the quasi-Fermi-level splitting^23^,^24^. Since we are more concerned on whether the photon-recycling effect is strong enough to impact the *V*_OC_ of perovskite solar cells, we focused on the measurement of emission intensity after multiple cycles of re-absorption and re-emission.

Materials with high photon recycling efficiency generally meet two criteria: (1) large overlapping of its PL spectrum with the absorption spectrum, that is, the materials should have a small Stokes shift or even an anti-Stokes shift, so that the self-absorption of light can be efficient; and (2) a high-internal PL quantum yield (higher than 80% for GaAs under strong illumination intensity of 100 sun)^25^,^26^. As demonstrated above, the re-absorption probability is very high in MAPbBr_3_ single crystals. Yamada *et al*.^27^ also observed a similar re-absorption phenomenon in MAPbI_3_ single crystals from a two-photon PL measurement. Such a large self-absorption ratio in perovskite materials meets the prerequisite for efficient photon recycling. It should be noted that the Stokes shift is usually positive for perovskite thin film, that is, the emission peak is red-shifted compared with the absorption edge^28^. The overlapping of the absorption curve and PL emission curve for a thick perovskite single crystal is purely a thickness effect, as thicker perovskite can absorb more band tail light, leading to a gradual red-shift of the absorption edge with the increase in perovskite thickness. Moreover, a high-PL quantum yield above 90% has been reported in some OIHP nanomaterials, which, however, does not make efficient solar cells because of a poor charge transport property^29^.

The photon recycling scenario was further promoted by the observation of unique double PL peaks in the PL spectra of many perovskite single crystals of different compositions^30^, despite the additional lower energy peak also assigned to the emission of excitons bound to the surface defects^31^ or the excess PbBr_2_ (ref. 32). Recently, Pazos-Outón *et al*.^22^ first reported experimental evidence for the photon recycling effect in lead triiodide perovskite polycrystalline thin films being that charge generation was observed in a region longer than 50 μm away from the light absorbing region. Though this length has not reached the reported longest carrier diffusion length in single crystal perovskite^22^,^33^, it is far beyond the diffusion length of polycrystalline films along the lateral direction where a lot of grain boundaries present.

In this study, we quantify the photon recycling effect in perovskite materials with their single crystals by directly measuring its efficiency. The origin of the double peaks in the PL spectra of a variety of perovskite single crystals is also investigated to determine whether they are caused by the photon recycling effect. The photon recycling efficiency in perovskite single crystals is quantitatively evaluated by measuring the ratio of the re-emitted photons to the incident exciting photons, which are differentiated from each other by modulating their polarization. The results reveal that the photon recycling efficiency is below 0.5% in both MAPbBr_3_ and MAPbI_3_ single crystals under light excitation intensity close to one sun, which excludes its contribution to carrier transport in perovskite single crystals.

## Results

### Quantifying the photon recycling efficiency

**Figure 1:**
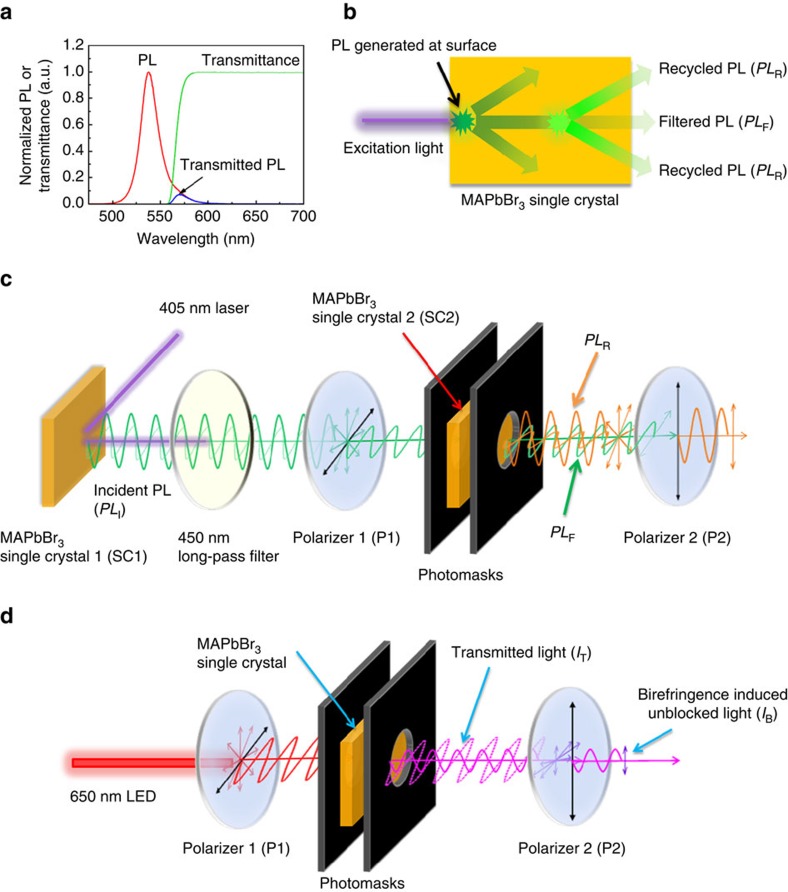
Scheme of the photon recycling measurement method. (**a**) Normalized photoluminescence (PL) spectrum of a 1 mm thick CH_3_NH_3_PbBr_3_ (MAPbBr_3_) single crystal (SC) measured with photomask (red curve), its normalized transmission spectrum (green curve), and the calculated transmitted PL spectrum (blue curve) by multiplying the PL spectrum by the transmission spectrum; (**b**) The schematic drawing of the photon recycling process in perovskite single crystals; The PL excited on the surface of the single crystal is absorbed during its transmission through the crystal and re-excite photons again, so that the emission from the bottom of the single crystal includes both the filtered photons (*PL*_F_) and recycled photons (*PL*_R_); (**c**) The schematic diagram of the measurement setup of photon recycling efficiency in perovskite single crystals; The *PL*_R_ and *PL*_F_ components can be differentiated based on their polarization difference, and the ratio of *PL*_R_ to incident PL (*PL*_I_) defines the photon recycling efficiency; (**d**) The schematic diagram of the measurement setup to determine the ratio of optical birefringence induced unblocked *PL*_F_ after transmission through perovskite single crystal by illuminating it with a 650 nm light emitting diode (LED); The intensity ratio of birefringence effect induced unblocked light (*I*_B_, when P1⊥P2) and transmitted light through the single crystal (*I*_T_, when P1||P2) defines the contribution of the unblocked *PL*_F_ to the *PL*_R_ spectrum.

We directly measured the photon recycling efficiency in perovskite single crystals based on the mechanism schematically shown in Fig. 1(b). After PL is generated on the top surface of a single crystal by the short wavelength light excitation, half of the PL emission transmits through the crystal and is filtered by the crystal itself. In addition, during its transmission, the high-energy portion of the PL is absorbed by the crystal itself, which yields re-emission with the PL peak shifting with respect to the absorption edge defined by the Stokes shift. Therefore, PL emitting out from the bottom of the crystal may be composed of both the filtered PL (*PL*_F_), and PL generated by multiple cycles of self-absorption and re-emission, that is, recycled PL (*PL*_R_). By measuring the ratio of *PL*_R_ and *PL*_F_ with respect to the total transmitted PL (*PL*_T_), one can determine the photon recycling efficiency in the single crystals.

We developed a method to separate these two types of PL emissions, with the setup schematically shown in Fig. 1(c). We used the surface PL emission from one perovskite single crystal (SC1), excited by a 405 nm laser with light intensity close to one sun, to excite the other single crystal (SC2). This mimicked the PL excited by a short wavelength laser at the SC2 surface but allowed us to accurately know the emission spectrum and excitation light intensity from the surface of the SC1. A long-pass filter with a 450 nm cutoff was inserted between SC1 and SC2 to eliminate the scattered 405 nm laser light. During the transmission of PL in SC2, it was partially absorbed by SC2 and caused re-emission (photon recycling). To exclude the influence of the light which leaked out from the edges of the SC2, two photomasks were attached to its top and bottom surfaces, respectively, which effectively prevented the double PL peak formation, as explained below. When we modulated the polarization of PL from SC1 by a linear polarizer (P1), we expected it to retain this polarization after transmission through SC2. In contrast, the recycled PL was assumed to be nonpolarized. Therefore, these two types of PL could be readily differentiated from each other by a second linear polarizer (P2) with a perpendicular polarization direction to P1 (P1⊥P2), which was expected to completely block the *PL*_F_ while transmitting 50% *PL*_R_. If there was re-emission of a large amount of photons after re-absorption, we expected to see a similar shape and intensity of the P1||P2 spectrum and P1⊥P2 spectrum, as schematically shown in Supplementary Fig. 1(a). In contrast, if the intensity of the P1⊥P2 spectrum was much smaller than that of the P1||P2 spectrum (Supplementary Fig. 1(b)), it would indicate that only a small amount of photons were re-emitted after re-absorption and, hence, a low photon recycling efficiency.

It was found that the optical birefringence of the MAPbBr_3_ single crystal could slightly change the polarization of the transmitted light, which was evidenced by its polarized microscopy image^34^. Therefore, *PL*_F_ cannot be crossed out up to the polarizer's light extinction limit (0.05%). The transmission light spectrum for P1⊥*P*2 is actually the sum of *PL*_R_ and unblocked *PL*_F_. So the *PL*_F_ that has not been crossed out after transmission through P2 needs to be subtracted to more accurately determine *PL*_R_, which can be measured with the setup schematically shown in Fig. 1(d). Specifically, the emission of a 650 nm light emitting diode (LED), whose photon energy is far below the band gap of MAPbBr_3_, was used to replace the PL from SC1 to pass SC2. Since the below-bandgap light was not expected to induce the photon recycling effect in perovskite single crystals, the intensity ratio of the optical birefringence-induced unblocked light (*I*_B_) to the light transmitted through the single crystal (*I*_T_) represents the portion of the unblocked *PL*_F_.

We first verified that the polarization of PL from SC1 after transmitting through P1 was linearly polarized. As shown in Supplementary Fig. 2, the polarized PL emission from SC1 after transmission through P1 was almost completely blocked by the P2 (P1⊥P2), for a blocking ratio of about 99.95% which represents the upper limit for the polarizers used. Also, the polarization of the PL emission from SC2 was investigated with both the reflection and transmission mode measurements. In the reflection mode, the PL excitation and detection were on the same side of the single crystals; while for the transmission mode, the PL detection was on the opposite side of the crystals with the excitation, as schematically shown in Supplementary Fig. 3(a) and 4(a), respectively. The corresponding PL spectra shown in Supplementary Fig. 3(b) and 4(b), proved that the PL emission from SC2 was nonpolarized, despite the excitation light being 100% polarized. Therefore, half of *PL*_R_ can transmit through P2, as shown in Fig. 1(c).

**Figure 2:**
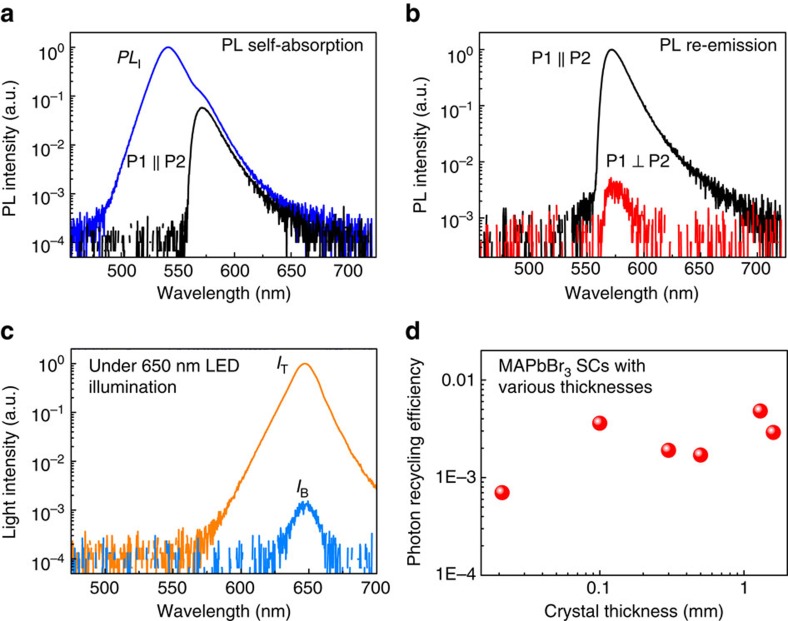
Photon recycling efficiency in MAPbBr_3_ single crystals (SCs). (**a**) *PL*_I_ (blue curve) and *PL*_T_ (black curve) spectra of a 1.3 mm thick MAPbBr_3_ single crystal showing its PL self-absorption ratio, where *PL*_I_ represents the incident PL from SC1 shown in Fig. 1(c), and *PL*_T_ is measured by adjusting the polarization direction of P1 and P2 in Fig. 1(c) to be parallel with each other; (**b**) *PL*_T_ and *PL*_R_ spectra showing the PL re-emission ratio, where *PL*_R_ is measured by adjusting the polarization direction of P1 and P2 in Fig. 1(c) to be perpendicular with each other; (**c**) The spectra of the 1.3 mm thick MAPbBr_3_ SCs excited by a 650 nm LED whose photon energy is far below the band gap of MAPbBr_3_, and measured with the polarization direction of P1 and P2 in Fig. 1(d) to be P1⊥P2 (blue curve, *I*_B_) or P1||P2 (orange curve, *I*_T_) in order to determine the ratio of optical birefringence induced unblocked PL after transmission through perovskite single crystal; (**d**) The summarized photon recycling efficiency of MAPbBr_3_ single crystals with different thicknesses.

We measured the spectra of *PL*_R_ and *PL*_T_ of a 1.3 mm thick MAPbBr_3_ single crystal to determine its photon recycling efficiency by adjusting the polarization direction of P1 and P2 to be P1⊥P2 or P1||P2, respectively. The MAPbBr_3_ single crystals used for the measurements were grown from solution by a modified antisolvent crystallization method^34^,^35^, and the high quality of these crystals was evidenced by excellent transparency, a record high mobility lifetime product, and the high sensitivity of the X-ray detectors made from them, as previously reported by us^34^. To improve the surface quality of the single crystals, a ultraviolet-ozone treatment was performed on them for 10 min, which has been proven to reduce surface recombination velocity to be comparable with the best passivated silicon^34^. As shown in Fig. 2(a), only the long wavelength tail of the incident PL (*PL*_I_) from SC1 transmitted through the SC2, regardless of the polarization direction of P2. The integrated intensity of *PL*_T_ is about 4.7% of that of *PL*_I_, corresponding to a large re-absorption ratio of 95.3%. The integrated PL intensity for P1⊥P2 was only 0.40% of that of *PL*_T_ (Fig. 2b), indicating most of the transmitted PL was contributed by the filtered PL. The portion of unblocked *PL*_F_ induced by the birefringence effect, which needs to be subtracted from the *PL*_R_ spectrum, can be obtained based on the *I*_B_/*I*_T_ ratio when using the emission of a 650 nm LED to penetrate the single crystal (Fig. 2c). It was noted that only the photons of *PL*_R_ emitted within the critical solid angle (escape cone, Ω) can escape the crystal through the bottom surface, which was calculated to be 0.3*π* based on the refractive index of MAPbBr_3_ (*n*=1.9) (ref. 36) and air (*n*=1) and assuming an isotropic emission of *PL*_R_. The light out-coupling ratio of *PL*_R_ was thus calculated to be 7.5%. So the actual integrated intensity ratio (*η*) of recycled PL to incident PL, or the photon recycling efficiency, was calculated to be 0.48% based on the equation below:


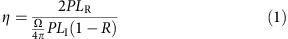


where *R* is the reflectance of the single crystal surface, and the ratio was multiplied by a constant of 2, because only half of *PL*_R_ can transmit through P2. We have also measured the photon recycling efficiency of several other pieces of MAPbBr_3_ single crystals with different thicknesses, and all of them exhibited a low efficiency below 0.5%, despite of a high re-absorption probability larger than 70%, as summarized in Supplementary Fig. 5 and Fig. 2(d). The above results indicate that the photon recycling effect is insignificant in MAPbBr_3_ single crystals under one sun excitation.

**Figure 3:**
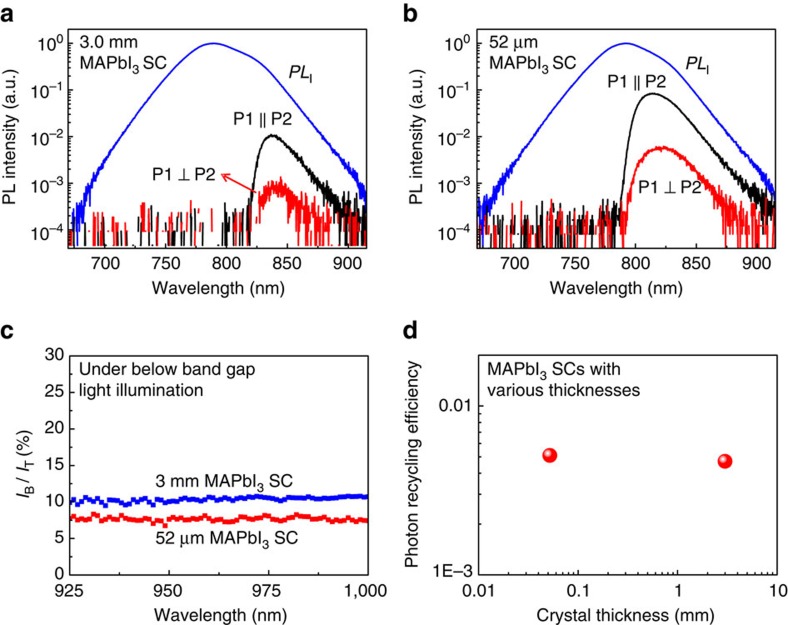
Photon recycling efficiency in CH_3_NH_3_PbI_3_ (MAPbI_3_) single crystals. (**a**,**b**) *PL*_T_ (black curve), and *PL*_R_ (red curve) spectra of a 3 mm thick (**a**) and a 52 μm thick (**b**) MAPbI_3_ single crystal (SC) measured by adjusting the polarization direction of P1 and P2 in Fig. 1(c) to be parallel or perpendicular with each other, respectively; The blue curve is the *PL*_I_ spectrum; (**c**) The *I*_B_ to *I*_T_ intensity ratio of the 52 μm (red symbol) and the 3 mm thick (blue symbol) single crystals, excited by light with energy far below the band gap of MAPbI_3_ in order to determine the contribution from the optical birefringence; The ratios are comparable to values calculated from the PL spectra, indicating the dominating role of optical birefringence in the *PL*_R_ spectra shown in **a**,**b**; (**d**) The summarized photon recycling efficiency of MAPbI_3_ single crystals with different thicknesses.

The photon recycling efficiency of a MAPbI_3_ single crystal has also been investigated by the same method. We chose MAPbI_3_ single crystals of two representative thicknesses for the measurement. One was a 3 mm thick single crystal that was demonstrated to possess the ultralong carrier recombination lifetime^33^,^37^, and the other was a thinner single crystal with a thickness of 52 μm. As shown in Supplementary Fig. 6, the blocking ratio of PL from MAPbI_3_ SC1 was only 98.1% after transmission through two polarizers with perpendicular polarization directions, which was limited by the light extinction ratio of the near infrared (NIR) polarizers used here. Figure 3a,b shows that the integrated intensity ratio of the *PL*_R_ to *PL*_I_ was much larger than that of the MAPbBr_3_ single crystals. However, from the *I*_B_ to *I*_T_ intensity ratio excited by light with energy far below the band gap of MAPbI_3_ (Fig. 3c), one can find that the high *PL*_R_/*PL*_I_ intensity ratio mainly originated from the birefringence-effect-induced unblocked *PL*_F_. After subtracting this portion, and also taking into account the light extraction ratio, the calculated photon recycling efficiency was 0.47 and 0.51% for the 3 mm and 52 μm thick single crystals, respectively (Fig. 3d). This is comparable to that of the MAPbBr_3_ single crystals, indicating a low photon recycling efficiency in the MAPbI_3_ single crystals.

### Origin of double peaks in photoluminescence spectrum

**Figure 4:**
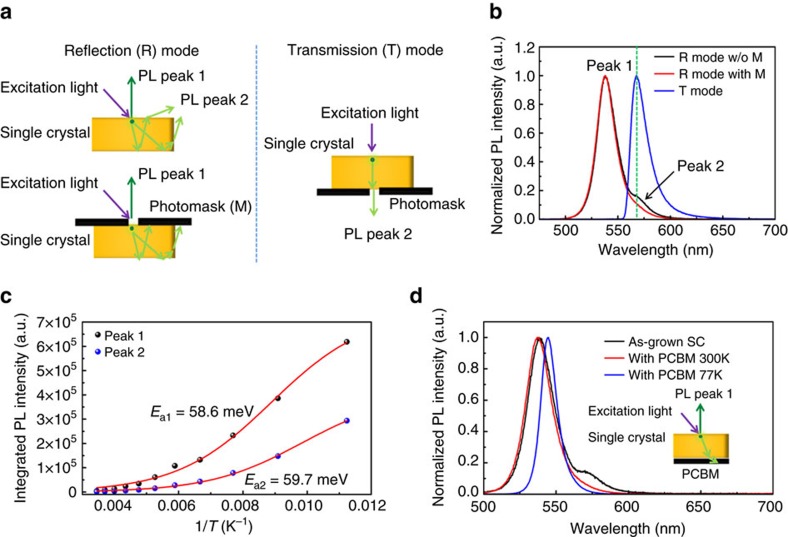
Origin of double peak in the PL spectrum of MAPbBr_3_ single crystals. (**a**) Schematic diagram of the PL measurement modes: reflection mode (left panel) and transmission mode (right panel); (**b**) Normalized PL spectra of a 1 mm thick MAPbBr_3_ single crystal (SC) measured under the reflection mode without photomask (black curve) or with photomask (red curve), and under the transmission mode (blue curve); The peak wavelength of the PL measured under the transmission mode matches that of the long wavelength peak (peak 2) measured under the reflection mode without photomask, as marked by the green dashed line; (**c**) Temperature dependent integrated PL intensity of peaks 1 and 2 shown in **b**; The red curves are the typical fitting of the data by equation (2) to determine their activation energies (*E*_a1_ and *E*_a2_); (**d**) Normalized PL spectra of a MAPbBr_3_ single crystal measured under the reflection mode without photomask (black curve), and with the coating a thick [6,6]-phenyl-C61-butyric acid methyl ester (PCBM) layer on the bottom of the crystal measured at room temperature (red curve) or at 77 K (blue curve); the inset is a schematic diagram of the PL measurement setup.

Another question which needs to be answered is what is the origin of the double PL peaks that are observed in a variety of perovskite single crystals. We measured the PL spectrum of MAPbBr_3_ single crystals with both the reflection mode and transmission mode and with the measurement geometry schematically shown in Fig. 4(a), so that we could distinguish the transited and scattered fluorescence. Figure 4(b) shows a typical PL spectrum of a 1 mm thick MAPbBr_3_ single crystal excited by a 405 nm laser measured in the reflection mode., which shows a small shoulder at around 570 nm (Peak 2) with the main peak at 538 nm (Peak 1). However, when a photomask was applied on the excitation surface of the crystal to block the emission from areas surrounding the excitation spot and from the crystal edges (Fig. 4(a)), Peak 2 was significantly suppressed (Fig. 4(b)). The PL spectrum of the same crystal under the transmission mode showed only one peak with the same peak position as Peak 2 (Fig. 4(b)). Since the PL captured in the transmission mode mainly consisted of the PL transmitting through the whole crystals, it underwent self-absorption; and hence only the long wavelength emission emitted out of the crystal, as shown in Fig. 1(a). Based on the above results, it was concluded that the double peak observed in the PL reflection mode was a combination of the PL generated on the top surface (Peak 1), as well as the filtered PL leaking out from the top surface and edge of the crystal after self-absorption and multiple reflection (Peak 2). This result excludes the origin of the photon recycling effect as the origin of Peak 2.

To verify the origin of these two peaks, we measured the temperature-dependent PL spectra of the MAPbBr_3_ single crystal from 89 K to 290 K under the reflection mode without applying the photomask (Supplementary Fig. 7) and the temperature-dependent integrated PL intensity of Peak 1 and Peak 2 were fitted by the equation below to derive their activation energies (*E*_a_):^38^


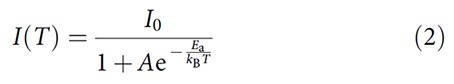


where *I(T)* is the PL intensity at temperature *T*, *I*_0_ is the PL intensity at 0 K, *k*_B_ is the Boltzmann constant, and *A* is a fitting parameter. As shown in Fig. 4(c), both fitted *E*_a_ of Peaks 1 and 2 were around 59 meV, indicating the same origin of the two peaks, instead of from the free and bound exciton emissions, respectively. To further confirm that Peak 2 was from reflection/scattering of the PL generated on the crystal surface, we dip coated a thick [6,6]-phenyl-C61-butyric acid methyl ester (PCBM) layer on the bottom of the crystals and measured the PL in the reflection mode without applying a photomask, as schematically shown in the inset of Fig. 4(d). We speculate that the coated PCBM eliminated the light reflection at the bottom surface by absorbing the PL. As expected, Peak 2 disappeared after coating with PCBM, both at room temperature and low temperature (77 K), as shown in Fig. 4(d). The disappearance of Peak 2 at low temperature also ruled out the possibility that Peak 2 originated from the defects-induced bound excitons because the relative intensity of the bound exciton peak in the PL spectrum was expected to be stronger at lower temperature.

## Discussion

**Figure 5:**
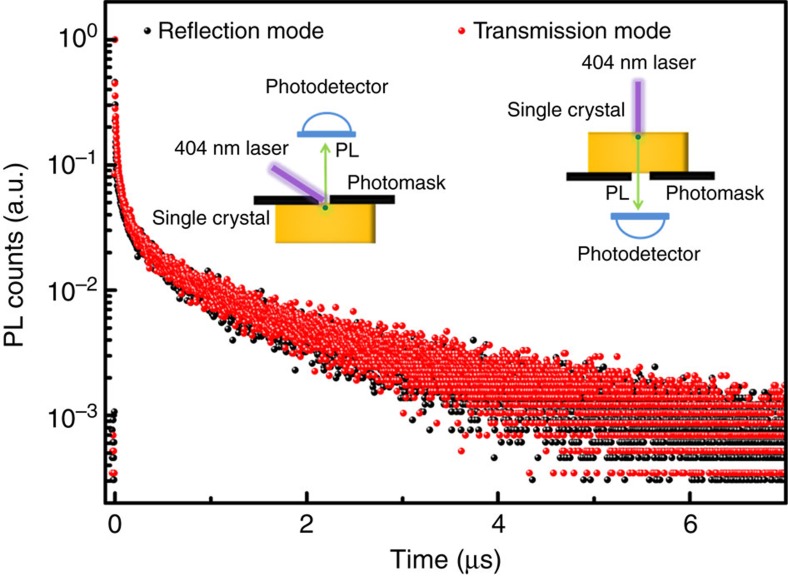
Time-resolved PL decay of a high quality MAPbBr_3_ single crystal. The time-resolved PL decay curves of a 3 mm thick high quality MAPbBr_3_ single crystal (SC) measured under reflection mode with photomask (black dot) and transmission mode (red dot); The inset shows the schematic of the measurement geometry of the reflection mode (left panel) and transmission mode (right panel); During the measurement, a 568 nm band-pass filter with band width of 10 nm was placed in front of the detector, in order to restrict the measurement wavelength range.

One of the direct consequences of the photon recycling effect was the elongation of the carrier radiative recombination lifetime because of the multiple cycles of regeneration of charge carriers by self-absorption^39^. We measured the radiative carrier recombination lifetime of a 3 mm thick high quality MAPbBr_3_ single crystal grown by a modified antisolvent crystallization method, which we recently demonstrated to show improved crystal quality and hence further increased sensitivity of X-ray detectors made of them (unpublished). This further verified the low photon recycling efficiency demonstrated above. The measurement was carried out both under reflection mode and transmission mode and with the application of a photomask. The PL measured under the reflection mode with a photomask mainly came from the crystal surface, while the PL measured under the transmission mode included both the filtered surface PL and the photon recycling PL. So if the photon recycling efficiency was high in the perovskite single crystals, the PL lifetime measured under the transmission mode would be much longer than that measured under the reflection mode. Since the PL lifetime of perovskite materials was wavelength dependent^40^, while the PL spectra measured under the reflection mode and transmission mode were quite different from each other, a 568 nm band-pass filter with a 10 nm band width was placed in front of the detector to restrict the detection wavelength range to be identical for these two measurement modes. As shown in Fig. 5, the PL decay measured under the reflection mode exhibited a fast decay within the first 100 ns, followed by slow recombination dynamics with a decay time constant of about 1.2 μs. The fast decay component may come from the carrier inward diffusion because of the high carrier mobility of single crystals^27^. Carrier recombination near the surface region may also have contributed to this decay process, while the slow component was generally assigned to the bulk recombination. The long decay time constant indicated the high quality of the single crystals. The PL decay curve measured under transmission mode almost overlapped with that measured with the reflection mode. This clearly indicates that the PL measured under the transmission mode was mainly from the surface-generated PL filtered by the crystal itself instead of the photon recycling PL. Hence its decay dynamics generally followed the trend of the surface PL decay process, which further confirmed that the photon recycling effect is not significant in these perovskite single crystals.

The low photon recycling efficiency of perovskite materials might be caused by its low PL quantum yield, which is reported to be highly dependent on excitation light intensity and is below 20% under one sun illumination at room temperature^22^,^41^,^42^. The light-intensity-dependent PL quantum efficiency was reported to be caused by a spin-split indirect-gap of perovskite material based on an *ab initio* relativistic calculation result. Its calculated radiative recombination rate was more than two orders of magnitude lower than that of GaAs and CdTe under one sun illumination^43^. Surface passivation of perovskite thin films could dramatically enhance the PL lifetime to be close to the intrinsic lifetime of single crystals^44^. However, the PL quantum yield of these passivated films with a PL lifetime longer than 8 μs was only 35%, which is still too low for efficient photon recycling. Future research is still needed to fully passivate the defects in perovskite materials for photon recycling purposes.

The low-photon recycling efficiency demonstrated in perovskite single crystals also indicates that their long carrier diffusion length, reported previously, is indeed because of other factors, such as its intrinsically high-carrier recombination lifetime, instead of the iterative absorption and re-emission process that increases the carrier transport distance. In fact, the ultralong carrier recombination lifetime of MAPbI_3_ single crystal has been verified by the time-resolved microwave conductivity technique^37^. During this measurement, the charge carriers are excited by light with a penetration depth comparable to the crystal thickness, so that the contribution from the photon recycling effect to the carrier lifetime is negligible.

Regarding the contribution of the photon recycling effect to the *V*_OC_ of perovskite solar cells, it is well known that the photon recycling effect can increase the *V*_OC_ of a solar cell by elongating the carrier recombination lifetime. Generally, the influence of carrier recombination lifetime on the *V*_OC_ can be estimated by the following equations:


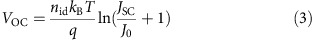



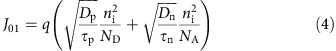



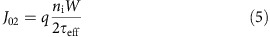



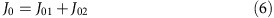


where *J*_SC_ is short circuit current, *J*_0_ is the reverse saturation current, *n*_id_ is the diode ideal factor, *J*_01_ is the diffusion-induced reverse saturation current, *D*_p,n_ are the diffusivity of holes and electrons, respectively, *τ*_p,n_ are the carrier lifetime of holes and electrons, respectively, *N*_D, A_ are the donor and acceptor concentrations, respectively, *n*_i_ is the intrinsic carrier concentration, *J*_02_ is the recombination induced reverse saturation current, *W* is the depletion layer width, and *τ*_eff_ is the effective recombination lifetime, or directly measured lifetime. Since the ideal factor for the perovskite solar cells is close to 2, we expect the *J*_02_ will dominate the reverse saturation current and assume *J*_0_=*J*_02_. The maximum photon recycling efficiency of 0.5% we measured in perovskite single crystals translates into an increase in the radiative recombination lifetime of only 0.5%. The corresponding *V*_OC_ increment was calculated to be only about 0.26 mV if the initial *V*_OC_ was assumed to be 1.1 V, which is basically in agreement with the measured no-change of PL recombination lifetime. For the current most emissive photovoltaic material, GaAs, it is reported that the radiative recombination lifetime could be increased by 4–6 times as a result of photon recycling^45^, which corresponds to a photon recycling efficiency of between 75 and 83%. However, its contribution to the *V*_OC_ enhancement in GaAs solar cells is still small. Even for the best GaAs solar cell, the open-circuit voltage increment as a result of photon recycling is only 4 meV under 1 sun condition^46^, probably because of the overwhelming nonradiative recombination in the current high-efficiency solar cells under operating conditions.

Note that the photon recycling efficiency measurement reported here was performed on perovskite single crystals rather than the polycrystalline thin films which are widely used in current high efficiency perovskite solar cells. It is not feasible to use it for photon recycling efficiency measurement, because the film thickness in polycrystalline solar cells is comparable to the light attenuation length, and thus re-emission/re-absorption does not occur as much in the out-of-plane direction. Nevertheless, since photon recycling in thin film devices refers to the light transport along the in-plane direction and/or across the film after multiple instances of reflection/scattering by the surface^22^, the light recycling across the single crystal is similar to thin film devices, from geometry aspects. On the other hand, the single crystals may exhibit a different PL property in comparison to the polycrystalline thin films, which means that the measurement results here cannot exclude the strong photon recycling efficiency in polycrystalline films. For instance, it was reported that the PL in CsPbBr_3_ might come from defects^47^, which could explain lower PL quantum efficiency in CsPbBr_3_ single crystals. Besides, the quicker carrier diffusion in single crystal samples as compared with polycrystalline thin films may also result in a lower PL quantum efficiency. However, we did not see obviously weaker PL from the MAPbBr_3_ single crystals as compared with the polycrystalline films, and the difference in reported results may be caused by an unawareness of the sensitivity of MAPbBr_3_ to environmental gases^48^. The perovskite grain size in the high-efficiency polycrystalline films was already over ten times larger than the exciton Bohr radius in these materials^49^, and thus the optical property of polycrystalline films approached that of the single crystals, though the photon recycling efficiency in polycrystalline films still needs to be determined by other independent characterization. Nevertheless, our results should still be relevant to the perovskite single-crystal-based solar cells, which have recently drawn increased research attention^50^,^51^ and may achieve comparable or better efficiency than the polycrystalline thin film based ones because of the orders of magnitude smaller trap density in single crystals. Finally, even if the photon recycling efficiency of perovskite polycrystalline thin films could be higher than that of the single crystals, its contribution to the *V*_OC_ enhancement would still not be significant with the present dominating device structures. The electron and hole transport layers always quench the PL from the perovskite films at a faster rate than PL emission, which results in a very low internal PL quantum yield. So the high *V*_OC_ of perovskite solar cells is mainly attributed to other factors, such as the unique defect-tolerant properties of perovskite materials^52^. In order to utilize the photon recycling effect to further increase the *V*_OC_ of perovskite solar cells, the nonquenching selective electrodes need to be explored to realize the high internal PL quantum yield and hence a high internal light intensity.

In summary, the photon recycling effect in perovskite single crystals was quantitatively evaluated by measuring the ratio of the recycled photons to the initially excited photons based on their polarization difference, which exhibited a low photon recycling efficiency <0.5% under one sun excitation intensity. The origin of the unique double peaks in the PL spectra of perovskite single crystals has been investigated in detail. It has been shown that the additional lower energy peak mainly comes from the filtered PL leaking out from the top surface and edge of the crystal after self-absorption and multiple reflections. The results presented here confirm that the long carrier diffusion length of perovskite single crystals previously reported is not facilitated by the photon recycling-induced photon propagation, highlighting their intrinsically excellent charge transport property.

## Methods

### Material synthesis

The MAPbBr_3_ thick single crystals were grown by the anti-solvent crystallization method^34^, and the MAPbI_3_ thick single crystals were grown by the top-seeded solution growth method^33^. The MAPbBr_3_ and MAPbI_3_ thin single crystals with thickness equal or <100 μm were grown from an ultrathin geometry-confined system^53^.

### Optical characterization

The transmission spectrum of the single crystals was recorded with a LAMBDA 1050 ultraviolet/vis/NIR spectrophotometer (PerkinElmer) equipped with an integrating sphere. The photoluminescence measurements were carried out on a Horiba 320 photoluminescence system with a 405 nm or 532 nm laser as the excitation light source. The temperature dependent PL measurement was performed in a temperature controlled probe stage with liquid nitrogen as the coolant. The polarization of the PL from MAPbBr_3_ single crystals was modulated with visible polarizers, while that from MAPbI_3_ single crystals was modulated with NIR polarizers. The time-resolved PL was measured with a Horiba DeltaPro time-correlated single photon counting system, and the 404 nm pulsed laser diode with pulse width of 45 ps was used as the excitation source. The scattered laser was eliminated with a 450 nm long-pass filter. And a 568 nm band-pass filter with band width of 10 nm was used to select the detection wavelength range.

### Data availability

The authors declare that the data that support the findings of this study are available from the corresponding author on reasonable request.

## Additional information

**How to cite this article**: Fang, Y. *et al*. Quantification of re-absorption and re-emission processes to determine photon recycling efficiency in perovskite single crystals. *Nat. Commun.* **8**, 14417 doi: 10.1038/ncomms14417 (2017).

**Publisher's note**: Springer Nature remains neutral with regard to jurisdictional claims in published maps and institutional affiliations.

## Supplementary Material

Supplementary Information Supplementary figures.
