# Supplementary material for: Quantification of re-absorption and re-emission processes to determine photon recycling efficiency in perovskite single crystals
Source: Nat Commun. 2017 Feb 21;8:14417. doi: 10.1038/ncomms14417 (PMC5321765; doi:10.1038/ncomms14417)
Supplement: Supplementary Information — Supplementary figures. [file ncomms14417-s1.pdf]

Supplementary Information

1. Schematic of the expected PL spectra of perovskite single crystals with high or low photon recycling efficiency

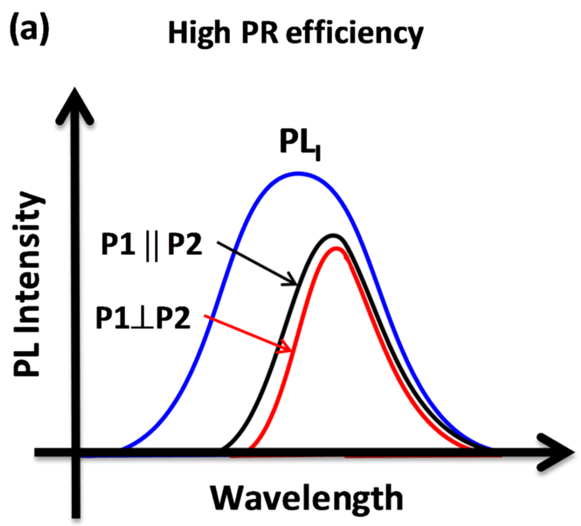

Supplementary Figure 1: Schematic drawing of the expected PL spectra of perovskite single crystals in the case of (a) high and (b) low photon recycling (PR) efficiency.

2. Blocking ratio of PL from $MAPbBr_3$ SC by visible polarizers

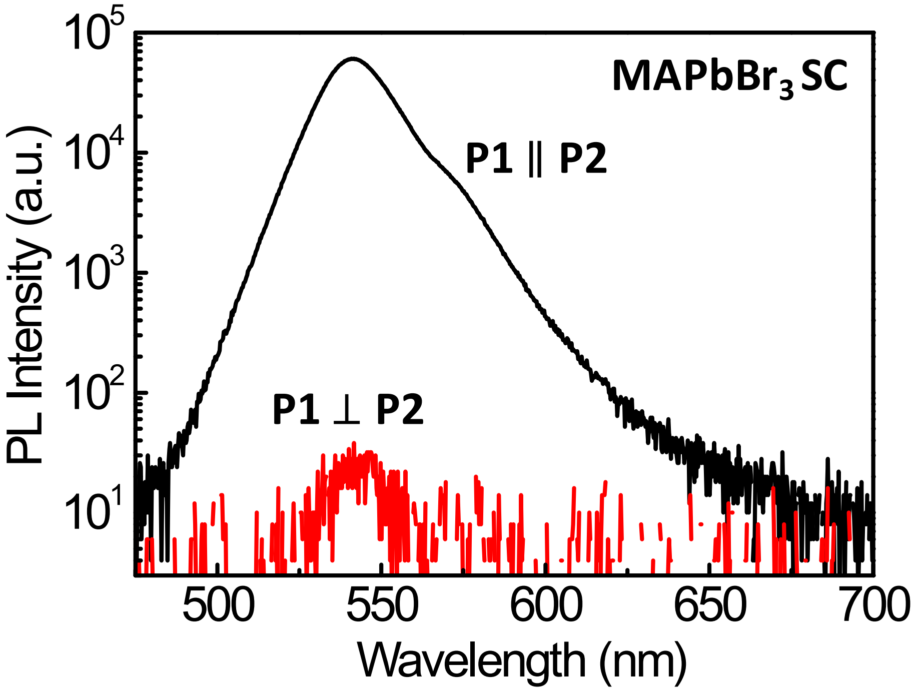

Supplementary Figure 2: The PL spectra of $MAPbBr_3$ SC1 measured by the setup shown in Figure 1(c) when the polarizer P1 and P2 are parallel or perpendicular with each other, and without $MAPbBr_3$ SC2. It is shown that the PL blocking ratio is about 99.95% by P1 and P2, limited by the visible polarizer itself.

3. Polarization property of PL from $MAPbBr_3$ SC excited by polarized light

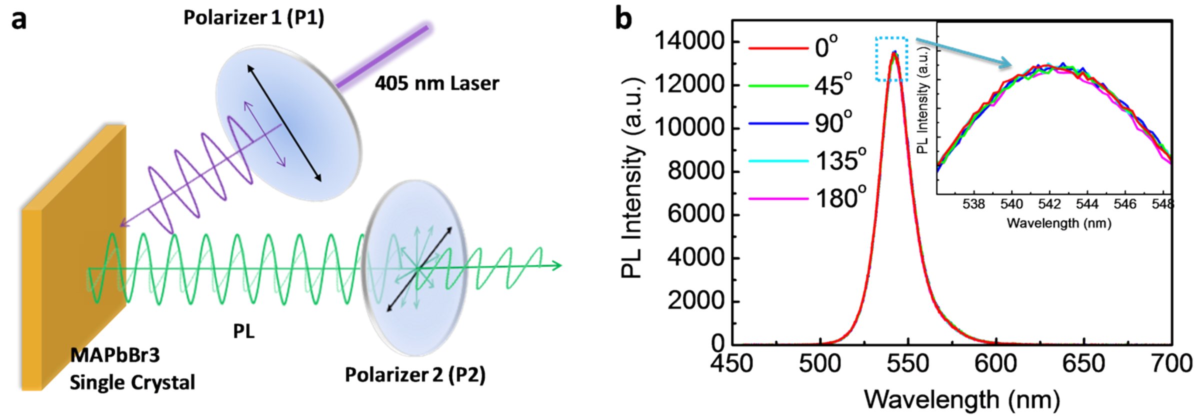

Supplementary Figure 3: The polarization property of scattered PL from a $MAPbBr_3$ SC surface excited by a polarized 405 nm laser. (a) Measurement setup; (b) PL spectra of the $MAPbBr_3$ SC measured under the reflection mode when rotating the polarization direction of P2 to be different angles with that of P1. It is shown that PL of $MAPbBr_3$ SC excited by a polarized light source is non-polarized.

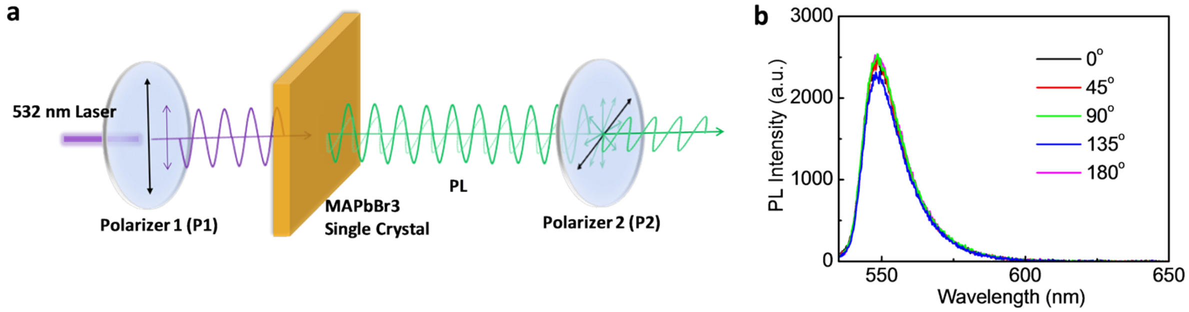

Supplementary Figure 4: The polarization property of transmitted PL of a $MAPbBr_3$ SC excited by a polarized 532 nm laser. (a) Measurement setup; (b) PL spectra of the $MAPbBr_3$ SC measured under the transmission mode when rotating the polarization direction of P2 to be different angles with that of P1. It is shown that transmitted PL of $MAPbBr_3$ SC excited by a

polarized light source is still non-polarized.

4. Quantifying the photon recycling efficiency of $MAPbBr_3$ SCs with various thicknesses

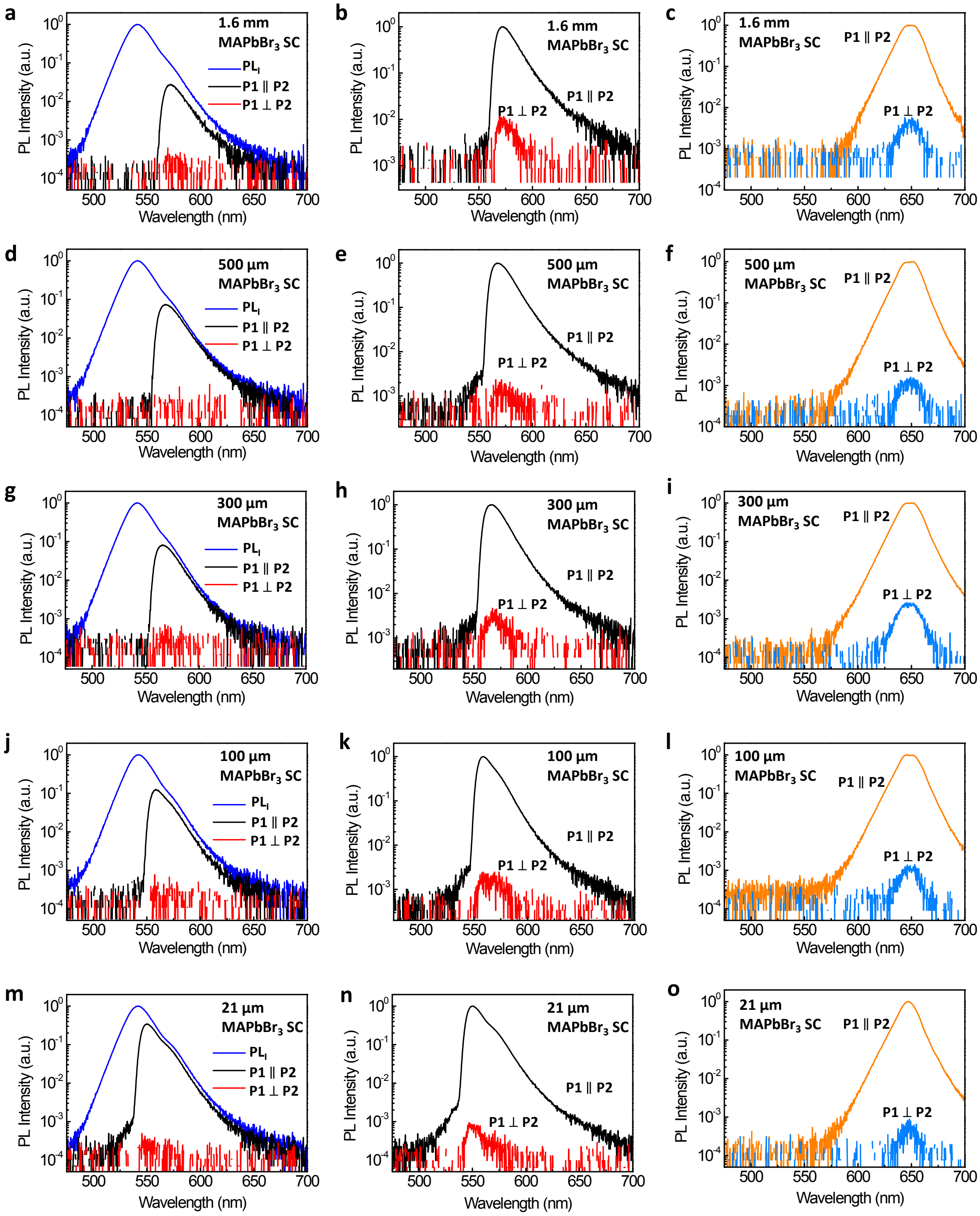

Supplementary Figure 5: (a, d, g, j, m) PL_T (black curve) and PL_R (red curve) spectra of $MAPbBr_3$ single crystals measured by adjusting the polarization direction of P1 and P2 in Figure

1(c) to be parallel or perpendicular with each other, respectively; The blue curve is the incident PL (PL_I) spectrum; (b, e, h, k, n) PL_T (black curve) and PL_R (red curve) spectra of the $MAPbBr_3$ single crystals measured with longer integration time to clearly show the PL_R spectrum; (c, f, i, l, o) The spectra of the $MAPbBr_3$ single crystals excited by a 650 nm LED measured with the setup shown in Figure 2(c), and with the polarization direction of P1 and P2 to be $P1 \perp P2$ (blue curve) or $P1 \parallel P2$ (orange curve), in order to determine the contribution from the optical birefringence to PL_R spectrum. The corresponding crystal thicknesses are: (a, b, c) 1.6 mm, (d, e, f) 500 μm, (g, h, i) 300 μm, (j, k, l) 100 μm, and (m, n, o) 21 μm.

5. Blocking ratio of PL from $MAPbI_3$ single crystal by NIR polarizers

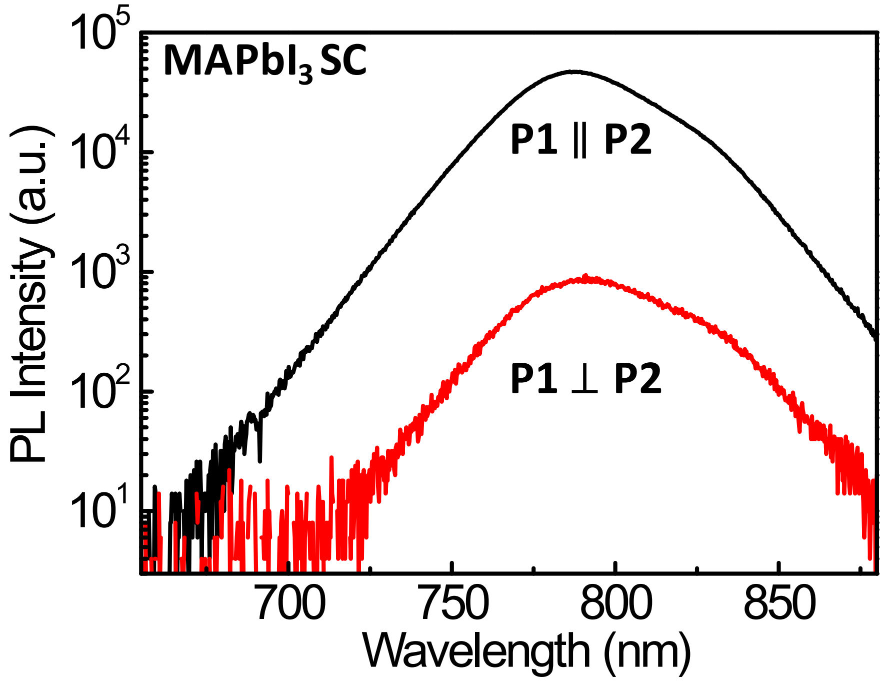

Supplementary Figure 6: The PL spectra of $MAPbI_3$ SC1 measured by the setup shown in

Figure 1(c) when the polarizer P1 and P2 are parallel or perpendicular with each other, and without $MAPbI_3$ SC2. It is shown that the PL blocking ratio is about 98.1% by P1 and P2, limited by the NIR polarizer itself.

6. Temperature dependent PL spectra of $MAPbBr_3$ single crystal

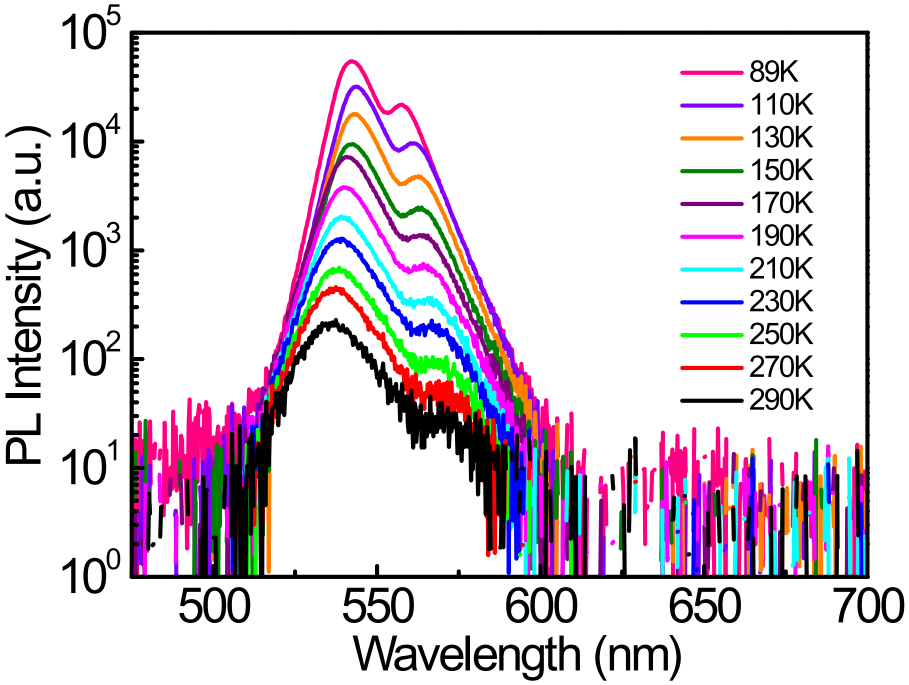

Supplementary Figure 7: Temperature dependent PL spectra of $MAPbBr_3$ single crystal excited by a 405 nm laser measured from 89 K to 290 K.
